# Why leaves become isotopically lighter than photosynthetic carbon isotope discrimination explains: on the importance of post-photosynthetic fractionation

**DOI:** 10.1093/jxb/erad497

**Published:** 2024-02-28

**Authors:** Arthur Gessler

**Affiliations:** Forest Dynamics, Swiss Federal Research Institute WSL, Birmensdorf, Switzerland; Institute of Terrestrial Ecosystems, ETH Zurich, Zurich, Switzerland

**Keywords:** Carbon isotope discrimination, phloem transport, post-photosynthetic discrimination, respiration

## Abstract

This article comments on:

Yu YZ, Liu HT, Yang F, Li L, Schäufele R, Tcherkez G, Schnyder H, Gong XY. 2024. δ^13^C of bulk organic matter and cellulose reveal post-photosynthetic fractionation during ontogeny in C_4_ grass leaves. Journal of Experimental Botany 75, 1451–1464

**Post-photosynthetic isotope fractionation needs to be considered in order to fully understand the information that is contained in the ‘isotopic fingerprint’ of plant organic matter. Yu *et al.* (2024) show that the isotopic composition of total organic matter in old leaves of a C**_**4**_ **grass does not necessarily reflect the photosynthetic processes that the carbon isotope signature is often assumed to record and integrate. This is due to post-photosynthetic carbon isotope fractionation which leads to a steady** ^**13**^ **C depletion in the overall dry matter of leaves over their life span. However, the authors show that leaf cellulose, which is laid down only at the beginning of leaf development, is a reliable proxy for photosynthetic parameters in the different water pressure deficit and N availability treatments they apply.**

## Post-photosynthetic processes affect the information that is contained in the isotopic fingerprint

While photosynthetic carbon isotope discrimination (Farquhar *et al.*, 1982) has become a process that is intensively studied to understand intrinsic water use efficiency in C_3_ plants and bundle sheath leakiness in C_4_ species, fractionating processes occurring in plant metabolism downstream of photosynthesis (Badeck *et al.*, 2005) have attracted much less attention. However, post-photosynthetic carbon isotope fractionation is of key importance as it alters the isotopic signature originally imprinted on the new assimilates during transport through plants and over time, complicating the interpretation of photosynthetic physiology (Gessler *et al.*, 2014). Even though recent studies pointed to the importance of post-photosynthetic carbon isotope fractionation at the leaf level (Gessler *et al.*, 2009) and provided more insight into the related processes (e.g. Lehmann *et al.*, 2019; Zhang *et al.*, 2019), we still lack quantifications of the carbon fluxes and the related isotopic fractionation into and out of the leaves to (i) obtain a proper isotopic mass balance and (ii) to assess the magnitude of the effects of post-photosynthetic processes on the isotopic composition of plant organs.

This is where Yu *et al.* (2024) make a big step forward by quantifying all major carbon fluxes of a leaf of the C_4_ grass *Cleistogenes squarrosa* and the fractionation associated with photosynthesis, leaf respiration, and export of assimilates. Based on these assessments, they develop a model to predict the overall isotope discrimination of leaf organic matter (Δ_DM_) and, thus, its isotope composition (δ_DM_). The central important observation by Yu *et al.* (2024) is that the observed Δ_DM_ increased (and thus δ_DM_ decreased) with leaf ontogeny, causing leaves to become isotopically lighter as they age. The output of their model accurately reproduces this ontogenetic pattern and thus allows the authors to identify that post-photosynthetic fractionation associated with respiration and with assimilate export is responsible for this.

Thus, continuous respiratory release of ^13^C-enriched CO_2_ and export of isotopically heavier carbohydrates to the phloem together lead to a cumulative ^13^C depletion of the organic matter over the lifetime of a leaf. This is certainly only true for metabolites with reasonable turnover and thus non-structural carbon, and the lack of any leaf-age related change in isotope fractionation of cellulose, which is only laid down in an initial phase of leaf development, proves this assumption.

## Temporal changes in the isotopic composition of leaf organic matter are related to organ-specific differences

It is also important to consider that this temporal ^13^C depletion of non-structural organic matter with leaf age is linked to organ-specific differences in the carbon isotope composition.

It has since long been known that there is a carbon isotope enrichment of heterotrophic tissues compared with leaves (Badeck *et al.*, 2005; Bowling *et al.*, 2008), as also seen in the study of Yu *et al.* (2024). Cernusak *et al.* (2009) have hypothesized six different mechanisms responsible for that, and respiratory fractionation in the leaves and assimilate export, namely the two central processes leading to the continuous ^13^C enrichment in leaves, are mentioned as two of them.

**Fig. 1.**
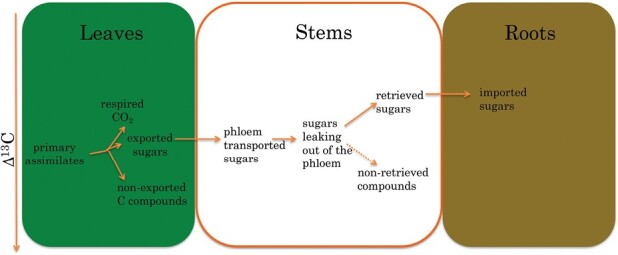
^13^C enrichment of carbohydrates transported from leaves to roots, and leaf-respired CO_2_ as related to the depletion of non-exported compounds.

The relative ^13^C enrichment of respired CO_2_ as well as of sugars exported from the leaves and its linkage to organ-specific ^13^C enrichment are depicted in Fig. 1. It needs to be noted here that leaf export and phloem loading do not discriminate *per se* but that they are rather the result of fractionations during metabolic conversions of the primary assimilates leading to compounds remaining in the leaves being ^13^C depleted. To conserve mass, the metabolites that are exported thus need to be ^13^C enriched. The isotopic fractionation observed by Yu *et al.* (2024) amounted to between –0.7‰ and –1.0‰, and between –0.5‰ and –1.0‰ for respiration and export, respectively. Numerically, the degree of depletion of the leaf total organic matter over time depends on respiratory and export fractionation weighted by the respective time-integrated fluxes in relation to the total leaf carbon pool.

Brandes *et al.* (2006) and Gessler *et al.* (2007) showed by measurement of phloem sugars that exported carbohydrates are ^13^C enriched compared with the non-structural carbon in the leaves (and also compared with the primary assimilates). As a consequence, the structural and non-structural carbon pools in the stems produced from these leaf-exported compounds will be isotopically heavier if we assume comparable metabolic processes as in the leaves with comparable fraction factors. Such ^13^C enrichment in newly produced stem wood compared with leaf organic matter has been observed in various C_3_ tree species (Gessler *et al.*, 2014; Gessler and Ferrio, 2022), and the findings of Yu *et al.* (2024) indicate that comparable organ-specific patterns can be seen in C_4_ species.

It has been observed previously that during basipetal phloem transport additional enrichment of the transported sugars occurred (Gessler *et al.*, 2007). The basis for the observed enrichment related to phloem transport is the dynamic Münch mass flow model (reviewed by Van Bel, 2003). The core of the model is the hypothesis that, while a proportion of the sucrose from sieve tubes is released during phloem transport, approximately two-thirds of it is retrieved and transported back into the sieve tubes (Minchin and Thorpe, 1987) where it is further transported in the direction of the sink tissues. In other words, the sieve tubes represent a system of ‘leaky pipes’ that lose and partially retrieve sugars during transport. The sugars leaking out of the phloem are subjected to metabolic conversions which leave the sugars reloaded in the phloem isotopically enriched whereas the sum of the non-retrieved compounds staying, for example, in the stem tissue are relatively depleted—but still enriched compared with the same pool in the leaves (Fig. 1). With such a sequence of sugar release, partial metabolic conversion, and retrieval during transport, the basipetal ^13^C enrichment of plant organic matter—that has its origin in the ^13^C depletion of leaves—can be described.

## New technological developments might provide new in-depth information on the metabolic processes involved in post-photosynthetic isotope fractionation

Yu *et al.* (2024) determined δ^13^C in bulk organic matter, cellulose in leaves of different ages, in primary assimilates and respired CO_2_ on the stand scale, as well as photosynthetic and respiratory fluxes. While they use this information in an intelligent way to elucidate overall integrating processes leading to the observed ^13^C depletion of organic matter in leaves, the detailed underlying metabolism still remains unexplored. New developments in soft ionization bioanalytical tandem mass spectrometers might open up new avenues for high-throughput compound-specific isotope analysis at natural abundance level (Neubauer *et al.*, 2023), and thus assessments of metabolic networks and fluxes and the related isotopic fractionations might become possible. Getting such insights would be the next step in our understanding of what information exactly is stored in the ‘isotopic fingerprints’ of particular chemical compounds and plant organs.
